# How to START? Four pillars to optimally begin your orphan drug development

**DOI:** 10.1186/s13023-023-02845-9

**Published:** 2023-08-03

**Authors:** Anneliene Hechtelt Jonker, Liliana Batista, Michela Gabaldo, Virginie Hivert, Diego Ardigo, on behalf of the IRDiRC ODDG TF and IRDiRC TSC

**Affiliations:** 1 IRDiRC, Paris, France; 2 TechMed Centre, University of Twente, Hallenweg 5, Enschede, 7522 NH The Netherlands; 3 Chiesi Farmaceutici S.p.A, Parma, Italy; 4 Fondazione Telethon, Milan, Italy; 5 EURORDIS-Rare Diseases Europe, Paris, France

**Keywords:** Orphan drugs, Drug development, Stakeholder analysis and engagement, Data gathering, Rare diseases, Patients’ needs

## Abstract

Drug development is a complex, resource intensive and long process in any disease area, and developing medicines to treat rare diseases presents even more challenges due to the small patient populations, often limited disease knowledge, heterogeneous clinical manifestations, and disease progression. However, common to all drug development programs is the need to gather as much information as possible on both the disease and the patients’ needs ahead of the development path definition. Here, we propose a checklist named START, a tool that provides an overview of the key pillars to be considered when starting an orphan drug development: STakeholder mapping, Available information on the disease, Resources, and Target patient value profile. This tool helps to build solid foundations of a successful patient-centered medicines development program and guides different types of developers through a set of questions to ask for guidance through the starting phase of a rare disease therapeutic pathway.

## Background

Rare diseases (RD) have become a priority in the political agenda over the past 20 to 30 years. The combination of regulatory incentives, public and private investment and a strong community of patients has paved the way for the growth of the research and development landscape for RD medicines, with several new drug approvals and blooming biotech’s pipelines [1–3]. However, a large majority of the 6000–8000 identified rare diseases remain ‘neglected’ in terms of medicines development, and even pre-development research on basic pathophysiology mechanisms and on clinical presentation/ natural history are lacking [4–7]. To help address the pressing need to develop medicines for patients with RDs, the IRDiRC Orphan Drug Development Guidebook (ODDG) was published in 2020 [8], proposing a roadmap to navigate in an efficient manner the available tools, resources and initiatives available to developers in the RD field. The Guidebook provides key recommendations tailored to the different stages of the drug development pathway. Both the Guidebook and the checklist are designed to begin with the end in mind, so to keep the patient’s need as a central point.

**Fig. 1:**
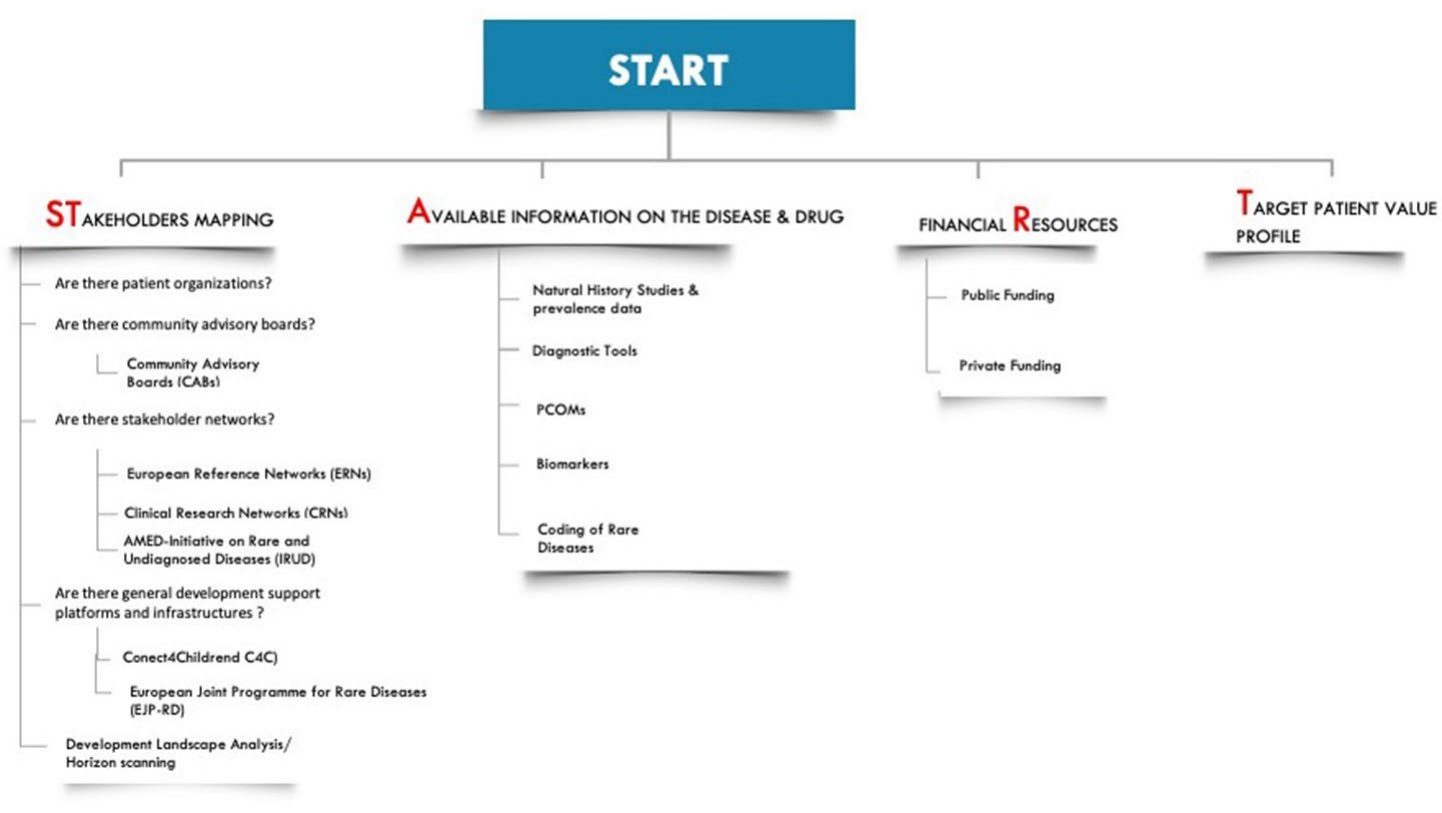
**START to drug development.** The different questions for each rare disease drug development project to ask are on Stakeholder mapping, thereby providing information on actors and fundamental infrastructure in the development process, Available information on the diseases, gathering information non the disease, financial Resources, gaining an overview on the financial means for the development and Target Patient Value Profile, before a stakeholder starts drug development. for Stakeholder mapping, this means looking at all stakeholders with a vested interest in the development process, such as patients, clinicians, and supporting contacts with platforms and development networks. For Available information on the disease, this entails searching for data and tools. For financial Resources, this means searching for a combination of public and private funding and setting out a fundraising strategy for the different steps in the development pathway. For the Target patient value profile, it means making an overview of the opinion and perspective of the patient regarding disease profile being investigated and the expected outcomes of a therapeutic development

One of the key findings of the project was the recurring need of starting to apply many tools very early in the development phase (even before a candidate drug is available) and to ground all future activities on pre-existing knowledge, resources, and stakeholder networks, which appear to be vastly lacking in these neglected disorders. To orientate the developer in this large portfolio of pre-requisites to be verified and of potential gaps to be urgently addressed at the start of the development path, we have generated a specific pre-development checklist, to be used as “starting blocks” of the development race. The four essential domains captured in the list are pictured by the acronym ‘ST.A.R.T.’, standing for: **ST**akeholder mapping, **A**vailable information on the disease, financial **R**esources, and the **T**arget patient value profile (Fig. 1).

The START checklist (Table 1) is relevant both in the context of better known and already well-researched diseases, but also - or even more - in the context of ‘neglected’, under researched conditions to help build the solid foundations of a successful patient-centered medicines development program. As such, the proposed checklist applies to all therapeutic areas in rare diseases, including all types of medicinal product modalities, for first-time regulatory approved products, for new active substance and products with or without an orphan designation. This tool, which is publicly available, can also be used by different types of developers for rare disease therapeutics, including but not limited to academic developers, small and medium enterprises, and patient-led developers[9]. This tool is applicable to any stage of a development program but with a focus on the pre-clinical elements. Whenever a developer starts the project, independent of the stage, they can use this tool to help make a strategic plan and use the checklist to revisit that plan over time.

**Table 1:** START Checklist

Question	Yes/ No	More information?
Stakeholder mapping		
Are there patient organizations for the disease?	□Yes □No	
Are there community advisory boards (CABs)?	□Yes □No	Community advisory boards
Are there clinical stakeholder networks?	□Yes □No	Engagement with established research networks The NIH rare diseases clinical research networks Japan Agency for Medical Research and Development (AMED) – Initiative on Rare and Undiagnosed Diseases
Are there general development support platforms and infrastructures?	□Yes □No	Conect4Children European Joint Programme on Rare Diseases
Have you done a landscape analysis or horizon scanning?	□Yes □No	Horizon Scanning: Landscape analysis/ Stakeholder identification and engagement
Available information on the disease		
Are there Natural History (NH) Studies?	□Yes □No	Natural History studies
Are there diagnostic tools?	□Yes □No	Companion diagnostics
Are there patient-centered outcome measures (PCOMs)	□Yes □No	Development of Patient-Centered Outcome Measures
Are there biomarkers?	□Yes □No	Use of biomarkers in orphan drug development
Is there a coding for the rare disease?	□Yes □No	Coding of rare diseases: Orphanet nomenclature
Financial Resources		
Did you acquire different sources of public funding?	□Yes □No	European Commission funded programs and resources European Joint Program on Rare Diseases NIH funded programs and resources AMED funded programs and resources
Did you acquire different sources of private funding?	□Yes □No	Private funding
Target Patient Value Profile		
Did you make a Target Patient Value Profile?	□Yes □No	Target Patient Value Profile

## Main text: START

### STakeholder mapping

A Stakeholder map can be described as a representation of all external parties, such as patients, clinicians, and other experts, who have a relevant interest for and a potential influence on the project, how these actors are (inter-)connected, and how these external parties represent stakeholder and the organization they’re representing. For any development program, it is essential that the developer builds as the first step (and maintains) a solid stakeholder network, which includes patients, clinicians, regulators, HTA experts, data and platform experts and other experts as needed, as this can provide information, knowledge, guidance, and support, which may lead to the success or failure of development, registration, and patient access[10]. The START checklist guides the developer in the identification of the before-mentioned key stakeholders by asking a series of key questions focusing on the existence of and accessibility to patient organizations, community advisory boards, medical healthcare networks, and connects them to owners and curators of platforms and infrastructures. When combined, these stakeholders are an irreplaceable source of unwritten knowledge about disease and patient’s needs, a unique resource in the design and conduction of clinical trials, and a paramount aid in the interpretation of the final benefit/ risk and value of the product. In the relationship with regulators, there is the possibility to have an open dialogue on non-product specific topics, such as general issues, guidelines, and disease endpoints. This can be done via the official regulatory procedures, such as scientific advice or non-official channels, such as multi-stakeholder forums and congresses.

The list also pushes developers to continuously operate a horizon scanning of the development landscape, projecting their thinking to the future landscape that will be unfolded when the product will be used in clinical practice, and ensuring the suitable stakeholders are considered for involvement as development progresses. As such, the horizon scanning provides an overview of the collaborative expertise and efforts of the stakeholders, thereby highlighting the vested interest of all players in the development of an orphan drug.

Patient organizations have to be involved at the beginning of any drug development program. It is essential to map if there are any relevant patient organizations representing the disease, where these organizations are based and how they represent specific geographic areas, if there are any relevant community advisory boards already constituted and operating, what is their main expertise and scope. Networks of healthcare professionals, researchers and physicians are also invaluable, and include umbrella initiatives such as European Reference Networks in EU, Clinical Research Networks in the US or the AMED-IRUD initiative in Japan[11–13]. Finally regional initiatives like the European Joint Program for Rare Diseases or the Pediatric Clinical Research Networks C4C (Conect 4 Children) for pediatric trials in Europe can be relevant sources of support in the developments[14–16].

A historical and current development landscape analysis is necessary to identify who is working in a particular disease area. This is normally a competitive analysis for the industry to determine the commercial potential of the development, as well as the future place on therapy of the product. For academic developers, it may be relevant to avoid duplication, provide intelligence on successful or failed approaches to guide product discovery and development and to suggest potential collaborative work with other research groups.

Once identified, the key stakeholders must be engaged with a long-term plan for this activity to be continuously developed over time, throughout the entire drug development pathway. However, there are certain moments in time when these interactions and engagement are more important, and these correspond to key milestones of Product Development: (i) during the discovery to generate the Patient Target Value Profile (PTVP), a document that describes how new medicinal products will benefit to patients (ii) before entering First in Human Trial (iii) after the generation the first evidence of efficacy and safety (Proof of Concept), (iv) once the Pivotal data are available and (v) in some cases also when the product is about to undergo through the market access and reimbursement discussions. Despite these key milestones, more regular consultation with stakeholders can be necessary and helpful for the successful progression of the project.

### Available information on the disease

Rare diseases are rare by definition; thus, it is very important to learn as much as possible from the patients affected by a rare condition, or to extrapolate from diseases with a similar etiology. The limited knowledge of the history and progression of the disease is typically one of the biggest challenges many developers have to face when developing medicines to treat a rare condition[17]. About 80% of rare diseases are genetic disorders, and the majority has a broad range of clinical heterogeneity. Such heterogeneity has an impact on determining meaningful outcomes that effectively address patients’ unmet needs and the interpretation of the data generated. Understanding the natural history of rare disorders is essential to understand the disease course and how it can be variable across patients, ensuring that appropriate clinical trial endpoints are incorporated in the trials. Depending on the information available on the disease, gaps in information need to be identified, so that additional information can be gathered if needed.

A disease natural history (NH) study) collects information about the clinical course, presentation, and progression of that disease. NH studies can be both retrospective by collating medical records and any other already available sources of information, or they can be prospective by collecting specific data to inform objectives of a study, often referred as a registry study. Detailed NH studies are also used to create historical and external control arms that can be used to evaluate the efficacy and safety of new treatments, resulting in an important alternative to placebo-controlled, when it is unethical or not feasible to do so[18]. Such NH studies can also be used in pharmaco-economic analysis to define the socio-economic burden of the untreated rare disease or later to develop a drug value proposition at the time of the market access.

For these reasons, the existence of a reliable source of NH data must be retrieved as soon as possible in development, or a solid NH evidence generation plan must be in place, potentially capitalizing on the partnership with relevant stakeholders. This is particularly relevant as real-world data (RWD), and real-world evidence (RWE) can also be leveraged in defining the NH studies of rare conditions. RWD and RWE are data collected through electronic health records by healthcare providers while providing their routine services. Clinical evidence generated from the analysis of these data can help to provide meaningful insights as to how patients fare in the real world with or without treatment.

Another critical element in enabling the progression of a drug development program is the presence of a clear diagnostic path and test(s) for the disease of interest, as well as tools for patient’s stratification (i.e., genotyping or phenotyping) and monitoring of response to therapy. If product-specific, companion diagnostic assays are only developed in parallel to a therapeutic product, or even after the product has been developed, and an inaccurate diagnostic test can lead to an incorrect treatment decision.

The success of such co-development also depends on the existence of specific biomarkers. The identification of biomarkers must pre-date drug development or occur during the very early research and preclinical phases, and this requires a thorough molecular understanding of both the disease biology and the drug mechanism of action[19].

Within the scope of understanding the disease, there’s the emerging notion that drug development outcomes should be measured from a more patient-focused perspective, which has led to the concept of patient-centered outcome measures (PCOMs)[20]. PCOMs consist of capturing the patients’ opinions and understanding how they cope with their health challenges and associated healthcare. A patient’s assessment on the health status (unmet needs, quality of life and symptom) offers the potential to provide therapies that are more valuable and aligned with patient’s priorities. It is not common in rare diseases that validated, or at least commonly accepted, PCOMs are already available and therefore the need for scouting relevant measures should occur at the beginning of the development journey, probably need to enter in the development of a new set of PCOMs or their extrapolation from other diseases. A logical step and questioning would be: has a PCOM been identified; if yes, does a corresponding measurement tool exist; if yes, has the measurement tool been endorsed by the regulators for use in clinical development?

Finally, one additional source to find disease information is through the Coding for Rare Diseases, a tool where a code is assigned each disease, so that it can be easily recognized within a health information system, such as healthcare databases and procedures related to patients’ cases and diseases. Such information is essential to make decisions on how to improve treatments, research, care and healthcare management. Currently there are several coding systems: the International Classification of Diseases (ICD) and is managed by the World Health Organization’s (WHO’s); the Systematized Nomenclature of Medicine Clinical Terms (SNOMED CT), run by the International Health Terminology Standards Development Organisation and is available in over 50 countries; and Orpha codes system that is based on Orphanet data and includes nearly 7000 rare diseases with a specific code, making it the largest coding system[21–24]. Overall, the above data sources will contribute to understanding the disease better and gaining a better understanding of the therapeutic need prior to starting the development.

### Financial resources: a continuous strategy

Drug development, for all diseases, including rare, is resource intensive, and the financial cost alone of developing a rare disease treatment has been estimated to be over 200 Million dollar[25]. In addition to the involvement of numerous partners throughout the development process, the quantity of investment often requires the involvement of multiple and heterogeneous financing mechanisms. It is therefore necessary to ensure incremental funding resources along the drug development pathway from the beginning to end, in order to develop the appropriate knowledge on the disease, to identify the appropriate technology which may address the respective underlying mechanism of action, to pursue the appropriate non-clinical and clinical development and to registration and launch of the drug. In other words, a developer must continuously look for funding until the drug is placed on the market and can be reimbursed.

With the key stakeholders and development requirements usually differing at each stage of development, this also implies that financing sources differ at each stage. Basic discovery research is funded primarily by public funding, such as local, national, and international governmental funding or funding by philanthropic organizations. Later stage development is funded mainly by private funding, such as funding by pharmaceutical companies or venture capitalists, while for the phase in between (basic research up to therapeutic development) hybrid financing mechanisms might be considered. Therefore, a sustainable financing strategy should be developed early on, to best make use of available funds, and financial sustainability may require a pluralistic approach in which needs are met throughout a combination of financing mechanisms, with no one-for-all strategy being available.

### Target Patient Value Profile (TPVP)

TPVP is proposed as a concept tool that gathers the input of the patients within the process of the generation of the optimal Target Product Profile (TPP)[26]. The TPVP is a concept we propose here, that would specifically capture the opinion and perspective of the patient regarding disease profile [27] and the expected outcomes of a therapeutic development. It is based on both the TPP which is a tool designed to outline the desired ‘profile’ or characteristics of a target product that is aimed at a particular disease or group of diseases (i.e., product labelling), and on the Target Development Profile, which is a tool to outline the desired profile, but also includes details on collaboration with all stakeholders[28]. It provides accurate, up-to-date information describing the expected benefit for patients. The TPVP should help to embed patient perspective across multiple domains: by understanding their needs, symptoms, and disease progression and how well the current standard of care meets their needs, what challenges they face and what are current therapy gaps. In other words, if developed and used appropriately, the TPVP can help developers to better plan and prioritize research methodologies toward filling up such patient identified gaps. Beyond the TPVP the engagement of the patients is a continuous process, and will be interwoven throughout the drug development timeline, but such engagement is particularly important prior to the start of therapeutic development to steer and set up research priorities. Overall, by having a TPVP that is co-designed with patients, we might be able to ensure that the TPP reflects the expected minimally acceptable product characteristics and desired value to be delivered to the most important stakeholder. Any developer is welcome to adopt the concept and adapt it to his/her own setting and situation.

## Conclusion

Although the popular motto “well begun is half done” might not necessarily apply to drug development, a significant proportion of factors with a relevant impact in the later stages are depending on pre-existing knowledge or decisions to be taken very early on in the development process. Examples of these are several previous papers have highlighted the importance of involving individual stakeholders and resources from the start, and the added value this represents, but also the additional costs it takes to later add aspects initially neglected. An example of these are studies that highlight the impact of patient engagement from the start, which leads to a faster enrolment of patient in clinical studies[29] or avoid protocol amendments [30], or overall bringing in the real-life experience for understanding the needs for developing orphan drugs[31]. A specific example from the Duchenne community describes the importance of stakeholder engagement needed to overcome the numerous challenges such as the developing the tools needed, and collecting relevant data[32]. In stages after the a successful start of orphan drug development, others highlight the increased compliance with procedures, and better use of regulatory incentives, resulting in a positive marketing authorization application[33, 34]. This is one of the crucial take-aways emerging from the IRDiRC’s ODDG, to guide the developer from the inception of a project, with the end in mind. To be successful, the design, including different innovative trial methodologies [35], conduction and interpretation of clinical trials in rare conditions, able to properly represent the real therapeutic value of a pharmaceutical product, require the mobilization or generation of vast informational resources and many of the supporting tools identified by the ODDG can provide valuable assistance only entering very early into play.

To highlight this concept and provide a valuable resource on day one of the research/ development project, we defined, based on the ODDG building blocks, a “starting blocks” checklist that is agnostic to the disease and to the medicine in development, using the acronym ST.A.R.T. START captures the optimal identification, connection, and engagement of four key pillars: **ST**akeholder mapping, **A**vailable information on the diseases, financial **R**esources, and **T**arget patient value profile. These four elements are the must-have departure point to build up the entire infrastructure around the development of a pharmaceutical product. By emphasizing the importance of these four pillars, our goal is to ensure that each drug development process gets off to a good start, potentially maximizing the speed of the development, and at the same time, reducing risk and costs, recognizing that it takes the responsibility of an entire community of stakeholders to generate and grow a valuable therapeutic option for our patients.

## Data Availability

N/A.

## Declarations

### Disclaimer

The views expressed in this article are the personal views of the author(s) and may not be understood as being made on behalf of their employ-er(s)/organization(s).

## Abbreviations

ICD: International Classification of Diseases
NH: Natural History
PCOMs: Studies, Patient-centered outcome measures
RD: Rare Diseases
RWD: real-world data
RWE: real-world evidence
ST: Stakeholder mapping
START: Available information on the disease, financial Resources, and the Target patient value profile
TPP: Target Product Profile
TPVP: Target Patient Value Profile
